# Minor grove binding ligands disrupt PARP-1 activation pathways

**DOI:** 10.18632/oncotarget.1742

**Published:** 2014-01-03

**Authors:** Kirill I. Kirsanov, Elena Kotova, Petr Makhov, Konstantin Golovine, Ekaterina A. Lesovaya, Vladimir M. Kolenko, Marianna G. Yakubovskaya, Alexei V. Tulin

**Affiliations:** ^1^ Blokhin Cancer Research Center RAMS, Moscow, Russia; ^2^ Fox Chase Cancer Center, Philadelphia, PA

**Keywords:** PARP-1, PARP-1 inhibitors, DNA-binding small molecules, poly(ADP-ribose)

## Abstract

PARP-1 is a nuclear enzyme regulating transcription, chromatin restructuring, and DNA repair. PARP-1 is activated by interaction with NAD+, DNA, and core histones. Each route of PARP-1 activation leads to somewhat different outcomes. PARP-1 interactions with core histones control PARP-1 functions during transcriptional activation in euchromatin. DNA-dependent regulation of PARP-1 determines its localization in heterochromatin and PARP-1-dependent silencing. Here we address the biological significance of DNA-dependent PARP-1 regulation *in vitro* and *in vivo*. We report that minor grove binding ligands (MGBLs) specifically target PARP-1 interaction with DNA, and, hence, the DNA-dependent pathway of PARP-1 activation. By obstructing its interaction with DNA molecules, MGBLs block PARP-1 activity *in vitro* and *in vivo*, as we demonstrate using *Drosophila*, as well as human cancer-derived cells. We also demonstrate synergistic inhibition of PARP-1, combining MGBLs with conventional NAD+-dependent inhibitors in human cancer cells. These results suggest that combining different classes of PARP-1 inhibitors can precisely modulate PARP-1 activity in living cells, thus holding promise for new avenues of cancer treatment.

## INTRODUCTION

**Figure 1:**
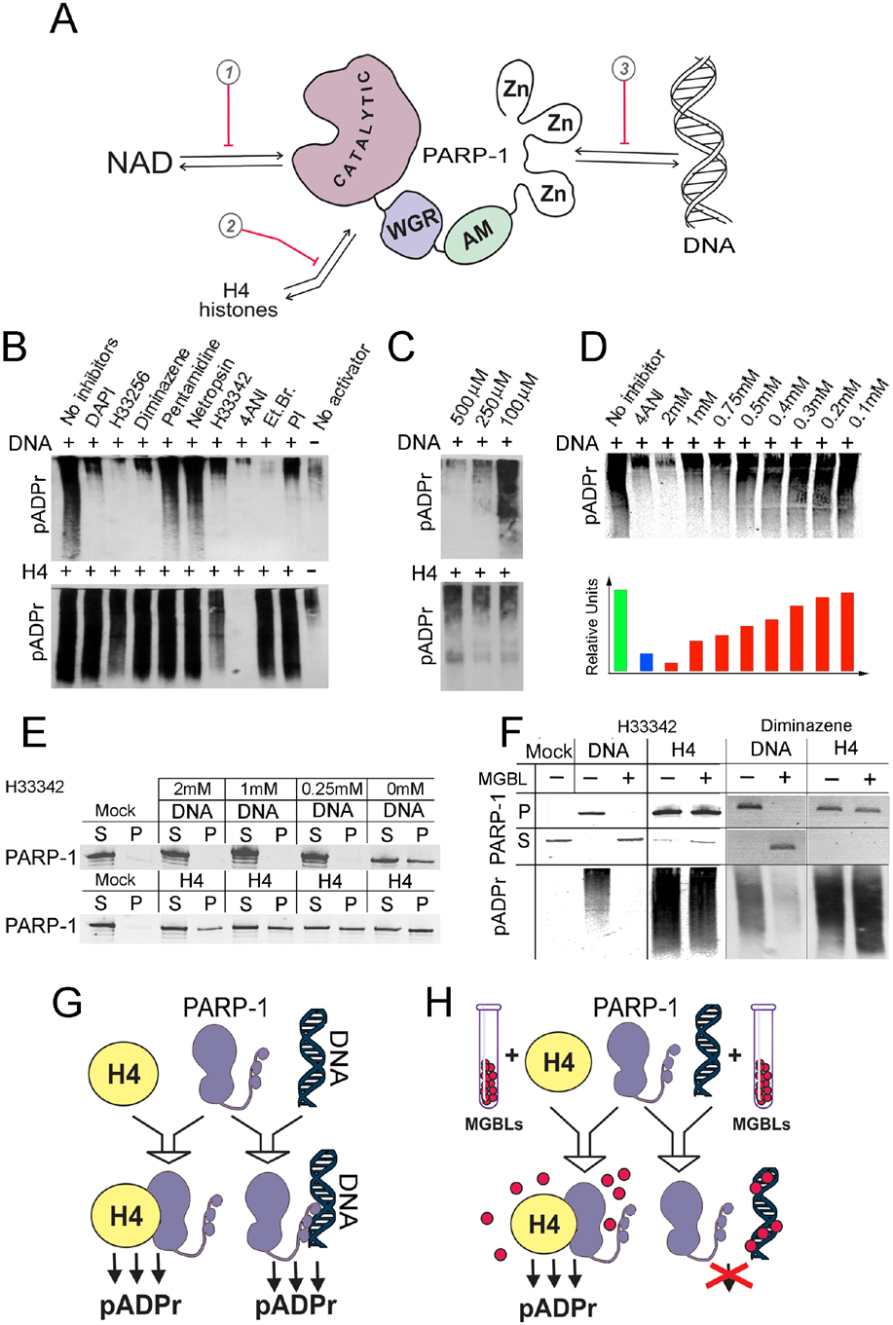
DNA-binding small molecules inhibit PARP-1 protein by blocking its interaction with DNA A. Schema illustrates three ways of PARP-1 regulation: 1) competing with NAD binding, which represents the current design method; 2) disrupting PARP-1 interaction with histones; and 3) disrupting PARP-1 interaction with DNA. B. DNA-binding small molecules inhibit DNA-dependent, but not histone H4-dependent, PARP-1 activation. DNA (top) or H4 reaction mixtures were preincubated with different DNA-binding compounds or NAD competitor 4ANI, followed by mixing with PARP-1 and NAD. Accumulation of pADPr was detected on Western blots. C,D. Minor groove binding molecule Hoechst^33342^ specifcally inhibits DNA-dependent PARP-1 activation (C). Panel D shows Western blot (top) and quantifcation (bottom) of amount of pADPr accumulation after PAPR-1 activation by DNA with and without NAD competitor 4-ANI or with dilution of Hoechst^33342^. E. Hoechst^33342^ disrupts PARP-1 binding to DNA (top), but not to histone H4 (bottom). Either DNA or histone H4 was covalently coupled to CnBr beads, pre-incubated with or without Hoechst^33342^, and incubated with PARP-1-containing solution. After precipitation of beads, pellet (P) and solution (S) were subjected to PAGE and Western blot. The presence of PARP-1 in pellet and solution was detected on Western blot. F. MGBLs inhibit PARP-1 via disruption of PARP-1 interaction with DNA. The binding-activation assay is shown. Sepharose beads with covalently attached DNA or H4 were preincubated with 0mM or 0.25mM solution of Hoechst^33342^ or diminazene, incubated with PARP-1, and mixed with NAD. The presence of PARP-1 and pADPr in pellet (P) and solution (S) was detected on Western blot after PAGE. G,H. Diagram illustrates how MGBLs hinder PARP-1 DNA-dependent activation by obstructing PARP-1 interaction with DNAs.

Poly ADP-ribose (ADPr) polymerase 1, PARP-1, is an essential protein involved in a wide range of cellular activities [1]. PARP-1 catalyzes the transfer of ADPr moiety onto protein acceptors or onto existing poly(ADP-ribose) chains by utilizing the glycolytic intermediate nicotinamide adenine dinucleotide (NAD+) as a source of ADPr [1–3]. Automodification of PARP-1 and its target proteins is accomplished by adding ADPr to glutamic residues [1,2]. The addition of ADPr polymers regulates the catalytic and DNA binding activity of PARP-1, as well as the cellular activity and localization of its target proteins. PARP-1 enzymatic activity is required for normal assembly of higher-order chromatin structures and the transcriptional activation of heat-shock-dependent, NF-kB-dependent, ecdysteroid-dependent, and ribosomal genes [4–6]. In clinical studies, inhibitors of PARP-1 have been shown to selectively eliminate tumor cells [7–9]. Therefore, PARP-1 inhibitors have recently found widespread use in the development of novel strategies for cancer treatment, and several PARP-1 inhibitors are currently undergoing phase I/II trials for FDA approval for treatment of tumors [10,11]. However, a number of clinical studies have reported setbacks in research on PARP-1-based anticancer therapies [12,13]. Most PARP-1 inhibitors have been designed to compete with NAD for a binding site on the PARP-1 molecule (Figure 1A). Since NAD is one of the most common cofactors involved in many eukaryotic pathways, this strategy resulted in the discovery of many nucleotide-like inhibitors that have a fairly low specificity to PARP-1 and also target other enzymatic pathways involving NAD and nucleotides as cofactors. Moreover, cancer cells tend to rapidly develop dynamic resistance against a single anticancer drug [14]. Thus, greater efficacy of PARP-1 inhibition could be attained by targeting multiple known routes of PARP-1 activation to develop combination therapies.

At least three points of PARP-1 protein control *in vivo* have been documented: 1) binding with NAD that serves as a substrate and a source of ADP-ribose for PARP-1 [1,2], 2) activation of PARP-1 via interaction with specific histones [15,16], and 3) activation of PARP-1 by interaction with DNA [1,2] (Figure 1A). The two pathways of PARP-1 targeting to chromatin and regulation involve DNA-dependent and H4-dependent PARP-1 activations [15,16]. The first pathway controls PARP-1 binding to inactive chromatin and regulation via the interaction of Zn-finger of PARP-1 with the DNA molecule [17]. Consequently, small molecules that compete with PARP-1 for DNA binding might be a significant nexus of inhibition. In support of this notion, a recent study suggested that at least some PARP-1 inhibitory compounds do not compete with NAD and, therefore, may act by inhibiting DNA-binding [18–20]. Since a number of different small molecules, known as minor groove binding ligands (MGBL), can influence DNA-mediated enzymes [21], we tested several of them for their ability to inhibit PARP-1 *in vitro*. Unlike intercalating DNA-binding molecules, which are extremely toxic and mutagenic [22,23], the minor groove binding small molecules from the Hoechst and diminazene groups do not induce point mutations or otherwise possess a very weak base-pair substitution potential [24–26]. As point mutagenesis is one of the main causes of drug resistance in cancer chemotherapy, non-mutagenic minor groove binding small molecules may show promise in future drug development [27]. Some of the compounds from this group are already in clinical use [28,29]. We demonstrated that these well-known minor groove binding small molecules may serve as potent PARP-1 inhibitors that target the DNA-dependent pathway of PARP-1 regulation. According to our findings, MGBLs prevent PARP-1 activation by competing for preferential binding sites on the DNA molecule. This mechanism of PARP-1 inhibition by MGBLs affords these molecules two possible types of application: as self-acting cytotoxic agents and as a component of combination chemotherapy preventing DNA repair by PARP-1, thereby facilitating DNA damage in cancer cells caused by other anticancer drugs [30].

## RESULTS

### MGBLs inhibit PARP-1 by blocking its interaction with DNA

We compared the effects of 6 different MGBLs on PARP-1 activation by DNA and by H4 *in vitro*. 4ANI, a NAD competitor, was used as a positive control for both types of PARP-1 activation. Ethidium bromide (EtBr), representing a small molecule possessing high affinity to DNA, was also included in the analysis. We observed a strong and selective inhibitory effect for DAPI, diminazene, Hoechst^33342^, Hoechst^33258^, and ethidium bromide, all of which blocked the DNA-dependent pathway of PARP-1 activation with remarkable specificity (Figure 1B). The more lipophilic Hoechst^33342^ exhibits significantly higher cell permeability than Hoechst^33258^ and possesses proapoptotic effect on cancer cells [30], Therefore, we first tested Hoechst^33342^ to examine its ability to regulate PARP-1 functions *in vitro*. Different dilutions of Hoechst^33342^ had a gradual effect on DNA-dependent PARP-1 inhibition, but no effect on H4-dependent PARP-1 activity (Figure 1C–D). Preincubation with Hoechst^33342^ (Figure 1E–F) or diminazene (Figure 1F) completely disrupted the physical interaction of PARP-1 with DNA, but not with H4, thereby abolishing PARP-1 activation by DNA, but not by the histone (Figure 1F–H). Such specificity prompted us to test the effects of MGBLs on PARP-1 function *in vivo*.

### MGBLs compete with PARP-1 for DNA-binding

**Figure 2:**
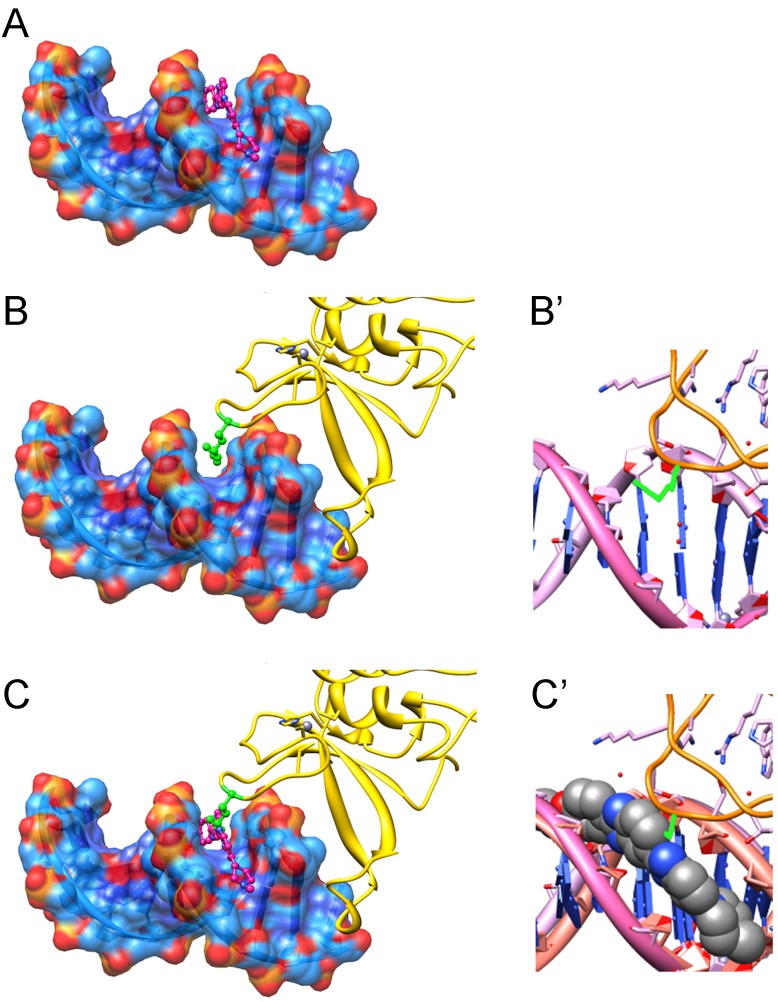
Model showing how PARP-1 protein competes with Hoechst^33342^ for DNA-binding (based on published crystallography data) The presence of MGBLs on DNA interferes with ZN-finger R122 intercalation between phosphor-sugar backbones in minor groove. Hoechst^33342^ docked to the minor grove of DNA duplex (according to PDB Code 129D) (A) and PARP-1 Zn-finger docked to DNA complex (B, B′) (according to PDB Code 4AV1). C, C' shows 45 A overlap between key Argenine 122 residue (green) of PARP-1 Zn-finger and Hoechst molecule.

Our data demonstrate that MGBLs specifically eliminate DNA-dependent functions of PARP-1. Moreover, our *in vitro* experiments showed that MGBLs inhibit PARP-1 by blocking the binding of PARP-1 to DNA molecules (Figure 1E, F), but not by directly interacting with PARP-1 or blocking PARP-1 interactions with other proteins (Figure 1E, F). To explore possible mechanisms of MGBL-mediated disruption of PARP-1-DNA interaction, we superimposed two previously reported crystal structures of Hoechst^33342^-DNA [37] (PDB Code 129D) and PARP-1 Zn-finger-DNA [38,39] complex (PDB Code 4AV1) (Figure 2A,B,B' and S1). As Hoechst^33342^ is known to interact with the central AT base pairs [30,40,41] in the duplex DNA, these base pairs were aligned and superimposed on the PARP Znf2 minor groove-interacting base pairs. As shown in Figure 2 and S1, binding of Hoechst^33342^ (magenta molecule) would preclude insertion of the key minor groove binding residue of Znf2, R122 (shown in green). Taken together, these data suggest that the presence of the minor groove binding dye would be expected to severely disrupt the binding of PARP-Zn fingers with DNA.

### The minor groove binding molecules Hoechst^33342^ and diminazene disrupt DNA-dependent PARP-1 localization and functions *in vivo*

**Figure 3:**
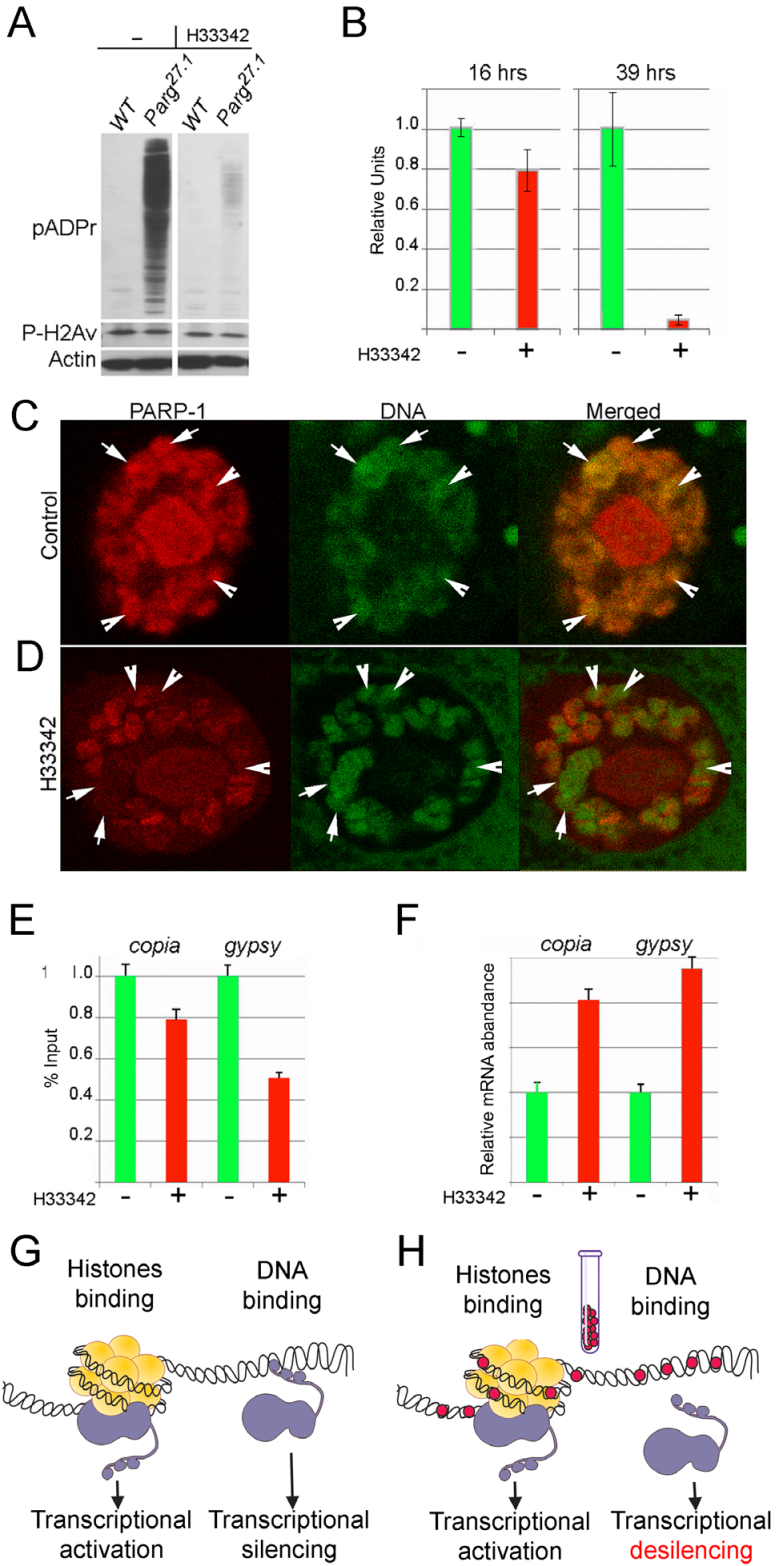
Minor groove binding molecule Hoechst^33342^ disrupts DNA-dependent PARP-1 localization and functions *in vivo* in *Drosophila* A-B. A comparative analysis of PAPR-1 protein activity in the *parg^271^* mutant third-instar larvae cultured with or without Hoechst^33342^ in the media. To detect pADPr on Western blot, mAb 10H antibody against pADPr was used. pAb antibodies against phosphorylated H2Av and Actin were used as a loading control. B. Quantification of pADPr accumulation in the *parg^27.1^* mutant third-instar larvae cultured with or without Hoechst^33342^ after 16 and 39 hrs of treatment. C-D. The treatment with Hoechst^33342^ disrupts Zn-finger 1-dependent PARP-1 localization in heterochromatin of *Drosophila*. Comparison of PARP-1 protein localization in salivary gland polytene nuclei in untreated control (C) and after 39 hrs of culturing with Hoechst^33342^ (D). PARP-1 is shown in red color and DNA in green. Constitutive heterochromatin is outlined with arrows. Arrowheads point to intercalary heterochromatin. E. Compared to wild-type untreated animals, PARP-1 protein binds chromatin of the heterochromatic elements *copia* and *gypsy* significantly less after culturing with Hoechst^33342^, as determined by ChIP assay. F. The quantitative RT-PCR assay shows that treatment with Hoechst^33342^ disrupts PARP-1-dependent silencing of the heterochromatic elements *copia* and *gypsy*. G,H. Diagram illustrating effects of MGBLs on DNA-dependent PARP-1 functions *in vivo*. G. In the absence of MGBLs, PARP-1 binding to DNA leads to transcription silencing, while PARP-1 interaction with nucleosomal histones leads to transcription activation. H. MGBLs obstruct PARP-1 binding to DNA, leading to desilencing of transcription; MGBLs have no effect on PARP-1 interaction with nucleosomal histones.

Unlike mammals with at least 17 PARPs, *Drosophila* has only one nuclear PARP, corresponding to human PARP-1 [4]. We recently demonstrated how DNA-dependent and histone-dependent functions of PARP-1 can be experimentally separated in *Drosophila* [17]. This makes the fruit fly invaluable in studying specifc functions of PARP-1. We therefore examined PARP-1 inhibition by Hoechst^33342^ in fruit fly. Precise measurement of pADPr levels in the wild-type fruit fly is complicated by the abundance of PARG protein, which rapidly cleaves pADPr *in vivo*, as well as during protein extract preparation [42]. To accurately measure pADPr levels, we performed inhibitory assays in the absence of endogenous PARG using *parg^27.1^* mutant animals [42]. Asynchronous *parg^27.1^* embryos and larvae were fed fruit fy food premixed with Hoechst^33342^ solution, and mature wandering third-instar larvae were collected after 16 or 39 hrs. When compared to wild-type animals at the same developmental stage, *Parg* mutant animals accumulated pADPr in a greater quantity (Figure 3A). However, culturing *Drosophila* in Hoechst-containing media significantly diminished the amount of pADPr detected (Figure 3A, B). Importantly, the agent approved in veterinary medicine, diminazene, showed a magnitude of PARP-1 inhibition in *Drosophila* similar to that of Hoechst (Figure S2A). The nucleoplasmic concentration of Hoechst and diminazene that was used during these experiments was significantly below saturation of their binding sites on DNA. Thus, these observations strongly suggest that MGBLs inhibit PARP-1 by competing with it for specific preferential binding sites on the DNA molecule, instead of nonspecifically obstructing PARP-1 binding to DNA by covering most of its length. These data confirm that MGBLs can function as potent PARP-1 inhibitors.

We next tested whether MGBLs could specifically affect DNA-dependent PARP-1 functions in fruit fly. Targeting of PARP-1 to the heterochromatin area of *Drosophila* genome and PARP-1-dependent transcriptional silencing are both controlled by the DNA-binding Zn-fingers of PARP-1 [17]. In wild-type *Drosophila*, PARP-1 has a broad, yet patterned, distribution along chromosomes, displaying considerable accumulation in regions of inactive, condensed chromatin (heterochromatin) with high DNA content (Figure 3B). Culturing wild-type *Drosophila* with Hoechst^33342^ (Figure 3C,D) or diminazene (Figure S2B) eliminates PARP-1 protein accumulation almost completely from “dense” chromatin, which corresponds to constitutive heterochromatin and intercalary heterochromatin, but it does not affect the binding of PARP-1 in decondensed loci that have low DNA content (Figure 3C–D; S2B). This observation suggests that specific DNA-dependent PARP-1 targeting to chromatin is inhibited by MGBLs.

We have previously shown that Zn-finger-dependent PARP-1 binding to heterochromatin is required for silencing of repeated heterochromatic DNAs [17]. Therefore, elimination of PARP-1 targeting to heterochromatin by Hoechst^33342^ should disrupt this silencing. We examined localization of PARP-1 in heterochromatin in Hoechst^33342^-treated animals and control using immunostaining of *Drosophila* polytene chromosomes and ChIP approaches. While PARP-1 in control animals shows significant accumulation on sequences of the retrotransposable elements *copia* and *gypsy* (typical content of silent chromatin), the amount of PARP-1 attached to these sequences is diminished after 39 hrs of Hoechst^33342^ treatment (Figure 3E). Moreover, we found that Hoechst^33342^-treated animals dramatically overproduce mRNA of both retrotransposons (Figure 3F). Thus, MGBLs appear to disrupt PARP-1 interaction with DNA in *Drosophila* and abolishes proper targeting of PARP-1 to heterochromatin, leading, in turn, to the desilencing of retrotransposable elements (Figure 3G,H).

### MGBLs synergistically interact with a classical PARP-1 inhibitor to block PARP-1 functions in human cancer-derived cells

**Figure 4:**
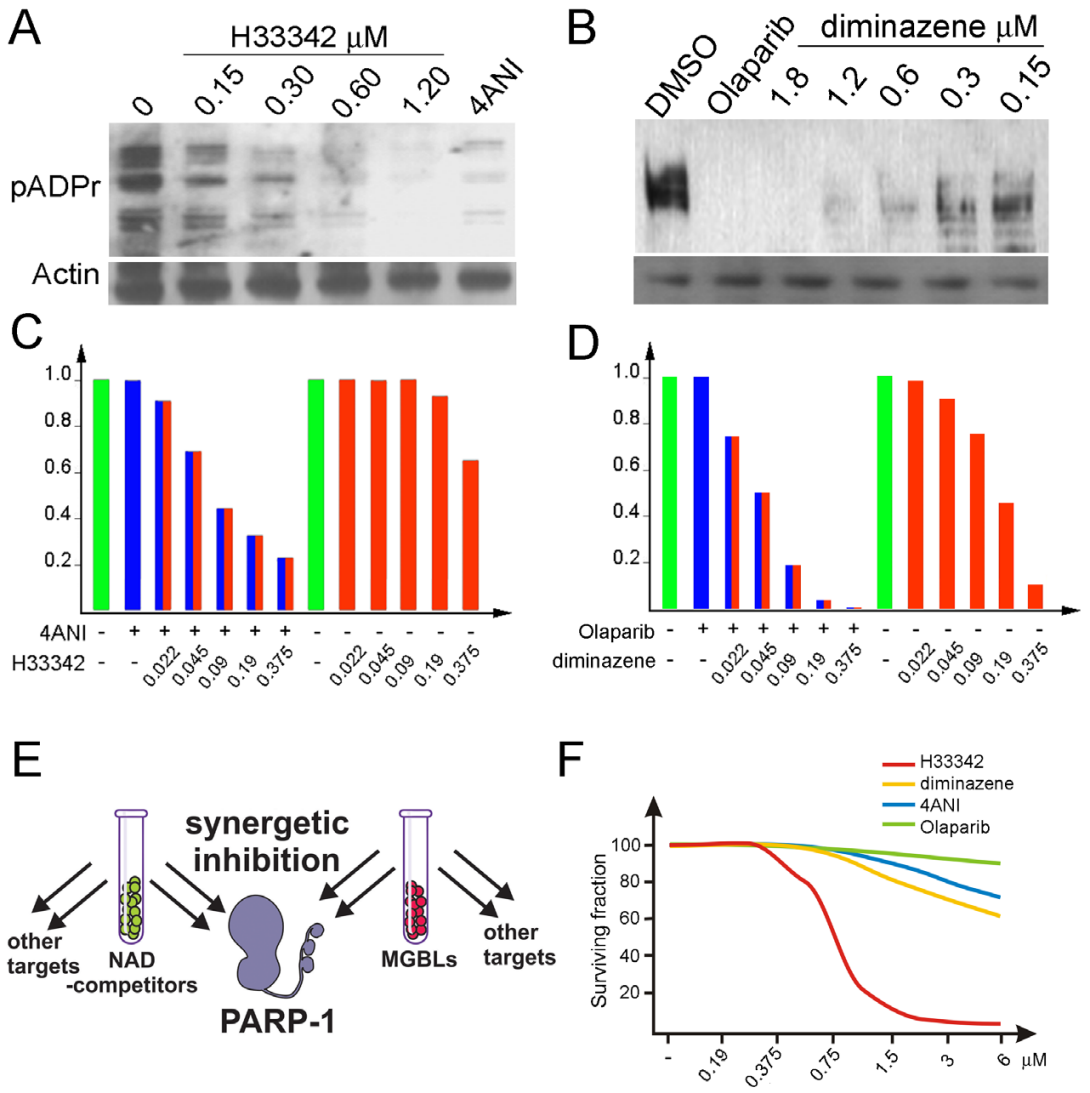
MGBLs inhibit PARP-1 activity in human cancer-derived cells A-B. A comparative analysis of PARP-1 activity in BT474 cells cultured with and without 4ANI and Hoechst^33342^ (A) or Olaparib and diminazene (B). To detect pADPr on Western blot, mAb 10H antibody against pADPr was used. pAb antibodies against Actin were used as a loading control. C-D. MGBLs synergistically interact with classical PARP-1 inhibitors to block PARP-1 functions *in vivo* in human cancer cells. (C) Quantification of pADPr amounts in BT474 cells treated with 4ANI and Hoechst^33342^ separately, as well as with combinations of 4ANI and different concentrations of Hoechst^33342^. (D) Quantification of pADPr amounts in BT474 cells treated with Olaparib and diminazene separately, as well as with combinations of Olaparib and different concentrations of diminazene. The amounts of poly(ADP-ribose), which reflect PARP-1 enzymatic activity, were detected after PAGE on Western blot using the anti-pADPr antibody and were quantified independently, using the Image Quant Software Package. E. Diagram illustrating the additive effect of MGBLs and NAD-competitors acting together. Apart from PARP-1, each of these chemicals targets other pathways. However, acting together, they are able to hinder PARP-1 activation via both routes with great efficacy. F. A cell viability assay shows the effects of BT474 cell treatments with 4ANI, Olaparib, diminazene and Hoechst^33342^ separately.

The efficacy of MGBL action on PARP-1 *in vitro* and *in vivo* suggests that MGBLs can be a starting point for developing novel drugs against PARP-1 to treat malignant tumors sensitive to PARP-1 inhibition. It has been shown that cells sensitive to PARP-1 inhibitors tend to have high preexisting levels of poly(ADP-ribose) [43]. Therefore, we tested Hoechst^33342^ using breast cancer-derived BT474 cells, which overaccumulate pADPr. Treatment of this cell culture with a NAD competitor, 4ANI (Figure 4A) or Olaparib (Figure 4B), diminishes pADPr amounts (Figure 4A). Similarly, Hoechst^33342^ (Figure 4A) and diminazene (Figure 4B) treatments block pADPr accumulation, suggesting that MGBLs are effective inhibitors of PARP-1 in human cells, just as in *Drosophila*.

To test the ability of DNA-dependent PARP-1 inhibitors to work synergistically with the classical PARP-1 inhibitor based on the competition with NAD, we compared PARP-1 activity in BT474 cells following dual and monotherapies with each reagent. Considerable decrease in PARP-1 activity in BT474 cells confirms that dual treatment with Hoechst^33342^ and 4ANI (Figure 4C) and/or with diminazene — Olaparib (Figure 4D) inhibits PARP-1 activity with significantly greater efficacy than either of these inhibitors applied separately (Figure 4C–E). Our findings demonstrate (Figure 4D) that both MGBLs and NAD-competitors are cytotoxic to human cancer-derived cells at very high concentrations only. Neither MGBLs nor NAD-competitors suppress the proliferation of cancer-derived cells, nor do they affect cell cycle progression (Supplemental Figures S3). Therefore, we further tested the ability of MGBLs and NAD-competitors to specifically suppress the tumorigenic potential of human cancer cells. For these experiments, we used the classical PARP-1 inhibitor Olaparib and the MGBL diminazene, both effectively inhibit PARP-1 *in vivo* at nanomolar concentrations.

**Figure 5:**
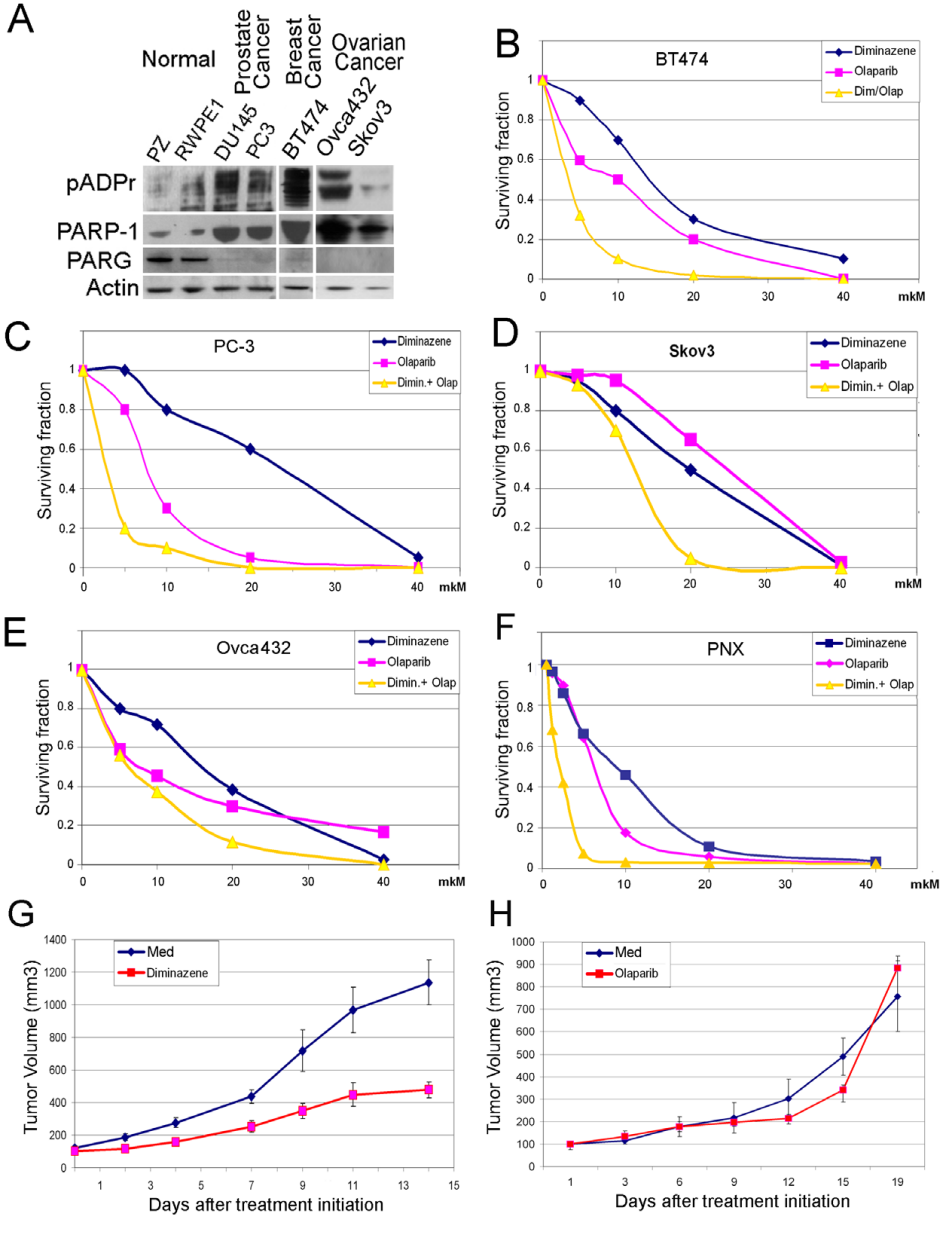
MGBLs suppress the growth of cancer cells Malignancy arising from cancer-derived cells is associated with aberrations in the regulation of pADPr turnover. Equal amounts of total protein extracts from normal (RWPE1 and PZ) and cancer-derived cells, including BT474 (breast cancer), DU145 and PC3 (prostate cancer), Skov3 and Ovca432 (ovarian cancer), were analyzed after PAGE on Western blots using anti-pADPr, anti-PARG, anti-PARP-1, and anti-Actin antibodies. Both cancer cell lines show defects in PARG expression and overproduction of PARP-1 and pADPr. B-F. Clonogenic Cell Survival Assay: BT474 (breast cancer) (B), PC-3 (prostate cancer) (C) Skov3 and Ovca432 (ovarian cancer) (D,E), PNX (renal cell carcinoma (RCC)). (F) Cells were plated into 24-well plates, allowed to adhere overnight and treated with increasing concentrations of diminazene (blue), Olaparib (magenta) and both (yellow) for 14 days. Colonies were counted and plotted on the graph. G-H. Diminazene inhibitor suppresses growth of patient-derived RCC xenograft tumors *in vivo*. Ectopic RCC xenograft tumors were established in 6-week-old male C.B17/Icr-scid mice using patient-derived PNX tumor cells. Animals were treated intraperitoneally with the MGBL diminazene (23 mg/kg) or vehicle (0.9% NaCl) (G), classical PARP-1 inhibitor Olaparib (Olap) (50 mg/kg) or vehicle (PBS + 10% (2-Hydroxypropyl)-b-cyclodextrin) (H) 5 days a week for 17 days (G-H). Values shown represent means (n=5) + SEM.

We compared the capacity of these small molecules to suppress the clonogenic potential of human cancer-derived cells. To accomplish this, we preselected cancer-derived cell lines [31–35], which, unlike normal human cells, demonstrate severe misregulation of pADPr pathway: BT474 (breast cancer), PC3 (prostate cancer), Skov3 and Ovca432 (ovarian cancer), as well as NKE and PNX (renal cell carcinoma (RCC) (Figure 5A). Cancer-derived cells express an abnormally high level of the PARP-1 protein and no PARG (Figure 5A). To examine the ability of diminazene and Olaparib to suppress the clonogenic potential [36] of human cancer-derived cells, we treated cells with these compounds separately and together. Although individual effects of Olaparib and diminazene vary from one cancer cell line to another, dual treatment with Olaparib and diminazene consistently demonstrated prominent synergistic efficacy in eliminating cancer-derived clones (Figure 5B–F). In light of encouraging *in vitro* data, we next examined the antitumor activity of non-NAD-like inhibitors using a renal cell carcinoma (RCC) xenograft tumor established from patient-derived tumor cells. As demonstrated in Figure 5G–H, animals treated with the MGLB diminazene showed a significant inhibition of tumor growth relative to control animals and animals treated with the classical PARP-1 inhibitor Olaparib. Importantly, treatment with diminazene was well tolerated by all animals, with no apparent signs of toxicity. These results are promising for the use of DNA-dependent PARP-1 inhibitors in therapeutic applications, when combined with conventional NAD-competing agents.

## DISCUSSION

By demonstrating an inhibitory effect of MGBLs on PARP-1, an enzyme required for DNA repair and, by extension, cell survival, we have explained the cytotoxicity of this compound that has been observed in a number of cell lines *in vitro* and in xenografts [27,44] (Figure 5G–H). According to our findings, MGBLs prevent PARP-1 activation by competing for preferential binding sites on the DNA molecule. This mechanism of PARP-1 inhibition by MGBLs affords these molecules two possible types of application: as self-acting cytotoxic agents and as a component of combination chemotherapy to prevent DNA repair by PARP-1, thereby facilitating DNA damage in cancer cells caused by other anticancer drugs [30,45,46].

Previously, MGBLs were shown to influence the activities of many DNA-processing proteins, such as topoisomerases, helicase, TATA box binding protein, replication protein A and others [30]. Most proteins that bind specifically to AT-rich DNA regions are considered to have extensive contact within the minor groove. Therefore, their inhibition should be mediated by direct steric hindrance [45,46]. Each MGBL binds AT-rich DNA in a sequence-specific manner, inhibiting a unique suite of DNA-binding proteins, thereby altering the pattern of protein activity and gene expression. Protein activity can be altered by at least two mechanisms: inhibition of specific genes and direct steric hindrance between MGBLs and DNA-binding proteins for specific binding sites on AT-rich DNA regions. An alternative mechanism of action by Hoechst^33342^ could involve changes to bent DNA conformations in genomic DNA. Since it has been suggested that 1) PARP-1 Zn-finger may preferentially interact with bent DNA [39] and 2) Hoechst^33342^ has been shown to mediate DNA bending [47,48], it seems plausible that an alternate mechanism of PARP-1 inhibition by Hoechst^33342^ could involve changes in the degree of DNA bending.

Because cancer cells multiply rapidly, any disruption of the household gene expression caused by MGBLs affects them more rapidly and to a greater extent than normal cells. As with other drugs, antitumor action of MGBLs is expected to be restricted by their toxicity to normal cells, in particular to hematopoiesis and the intestinal epithelium. However, unlike other drugs, MGBLs produce a unique profile of gene expression inhibition and protein synthesis that is specific for each MGBL. Therefore, MGBL-based drugs have a potential for selectivity against tumors in which mutations have established a distinct profile of household gene activity. Such MGBL-based drugs would have superior specificity and efficacy against sensitive tumorigenic cells, as suggested by other studies [40,41].

Taken together, the findings of this study lay the groundwork for the development of new small molecules directed against PARP-1/DNA activity, either as a monotherapy or in combination with known NAD-competing compounds or cytotoxic chemotherapies. New efficient and specific strategies for treating human malignancies based on PARP-1 inhibition will likely be developed in the future. More importantly, this study introduces a novel approach to designing PARP-1 inhibitors and proposes new strategies for eliminating tumorigenic cells and overcoming resistance to the NAD-competitive class of inhibitors.

## MATERIALS AND METHODS

### Human cell cultures

Human breast carcinoma cell line BT474 [31] was cultured in RPMI 1640 with 10% FBS, sodium pyruvate (10mM), N-2-hydroxyethylpiperazine-N'-2-ethanesulfonic acid (10mM) and antibiotics. Androgen-independent human PC-3 prostate cancer cells [32] were obtained from ATCC (Rockville, MD). Cells were cultured in RPMI 1640 (Bio-Whittaker, Walkersville, MD) supplemented with 10% FBS (Hyclone, Logan, UT), penicillin (100U/ml), streptomycin (100ug/ml), sodium pyruvate (1 mM) and non-essential amino acids (0.1 mM) under conditions indicated in the figure legends. Normal prostate epithelium cells RWPE-1 [33] were obtained from ATCC (Rockville, MD). RWPE-1 cells were maintained in Keratinocyte-Serum Free medium (Invitrogen, Carlsbad, CA) supplemented with 5 ng/ml of human recombinant EGF and 0.05 mg/ml of bovine pituitary extract. Ovarian cancer cell lines [34] were a kind gift from the Dennis Connolly lab. The NKE cells [35] were obtained from ATCC (Rockville, MD). The PNX cell line was a kind gift from Dr. Igor Astsaturov, MD, PhD (Fox Chase Cancer Center, Philadelphia, PA). Tumor cells were isolated from tumor tissue specimen obtained with written informed consent and Fox Chase Cancer Center Institutional Review Board approval (IRB approved protocol #12-822) from a patient undergoing tumor resection at the Fox Chase Cancer Center.

### PARP-1 inhibitory assay in human cell culture

Different doses of Hoechst^33342^, diminazene and Olaparib were added to the cells cultured in the complete medium. After 24 or 48 hrs, cells were lysed, and protein samples were analyzed with SDS-PAGE and Western Blot using anti-pADPr antibody.

### Treatment of Drosophila with MGBL PARP-1 inhibitors

WT flies were mass mated, and 10 females and 5 males were placed in vials with standard medium containing different doses of Hoechst (dose H10 – 330 μI of 10mM Hoechst^33342^ per each 8.25 g of medium; dose H20 — 330 μI of 20mM Hoechst^33342^ per each 8.25 g of medium) or distilled water. After 24 hrs, the parents were removed. Each experimental and control group consisted of four vials. In each vial, the number of pupae and imagoes were calculated and compared among the groups using Student's *t-*test.

### Clonogenic Cell Survival Assay [36]

Cells were plated into 24-well plates at a density of 2000 cells/well. Cells were allowed to adhere overnight at 37°C and treated with increasing concentrations of diminazene and Olaparib for 14 days. Colonies were fixed with 70% ethanol for 10 min and stained with 0.25% methylene blue in 30% ethanol for 10 min. After that, staining solution was removed, and plates were rinsed with water. Colonies consisting of 50 cells or more were counted. Plating efficiencies (PE) were calculated as follows: PE = number of colonies/number of cells seeded. The surviving fraction (SF) was calculated as follows: SF = number of colonies/number of cells seeded × PE.

### Ethics statement

This study was carried out in strict accordance with the recommendations from the Guide for the Care and Use of Laboratory Animals, as provided by the American Association of Accreditation of Laboratory Animal Care (AAALAC).

### Statistics

All data are presented as mean ± SEM. Statistical analyses were done using 2-tailed Student's *t*-test. A P value of 0.05 or less was considered significant.

## Supplementary Figures


