# Supplementary material for: Minor grove binding ligands disrupt PARP-1 activation pathways
Source: Oncotarget. 2014 Jan 3;5(2):428–37. doi: 10.18632/oncotarget.1742 (PMC3964218; doi:10.18632/oncotarget.1742)
Supplement: Supplementary file 1 [file oncotarget-05-428-s001.pdf]

SUPPLEMENTAL INFORMATION

Minor groove binding ligands disrupt PARP-1 activation pathways

Kirill I. Kirsanov[#1], Elena Kotova[&1], Petr Makhov[&], Konstantin Golovine[&], Ekaterina A. Lesovaya[#], Vladimir M. Kolenko[&], Marianna G. Yakubovskaya[#], Alexei V. Tulin[&]*

1 - These authors contributed equally to this work
& - Fox Chase Cancer Center, Philadelphia, PA 19111
# - Blokhin Cancer Research Center RAMS, Moscow, Russia

This file includes:

Supplemental Figures S1 to S3
Supplemental Materials and Methods
Supplemental References

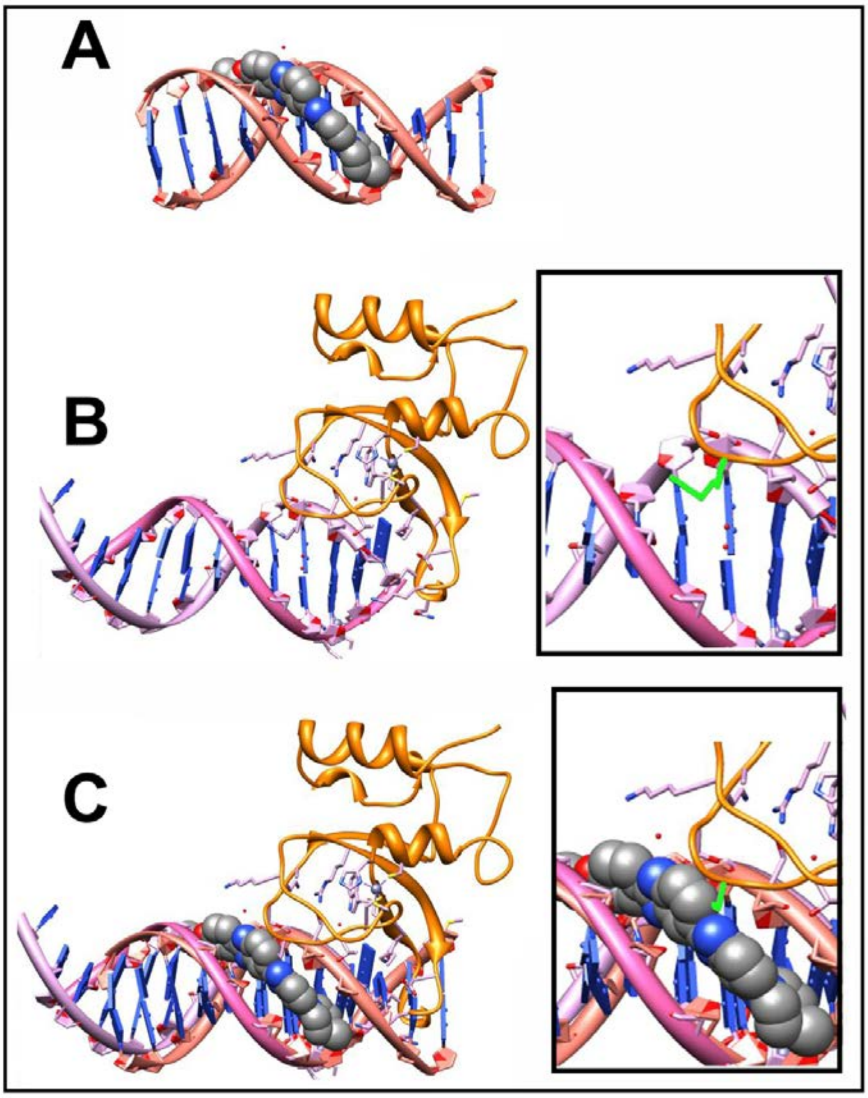

Figure S1. The molecular model based on published crystallography data showing how PARP-1 competes with Hoechst[33342] for DNA binding. MGBL bound to DNA hinders ZN-finger R122 intercalation with phosphor-sugar backbones in minor groove. **A.** MGBL molecule inside minor groove. **B.** ZN-finger R122 intercalation with phosphor-sugar backbones in minor groove. Right insert: magnification of the interaction region. **C.** MGBL molecule interferes with Zn-finger binding to phosphor-sugar backbones in minor groove. Right insert: magnification of the interaction region.

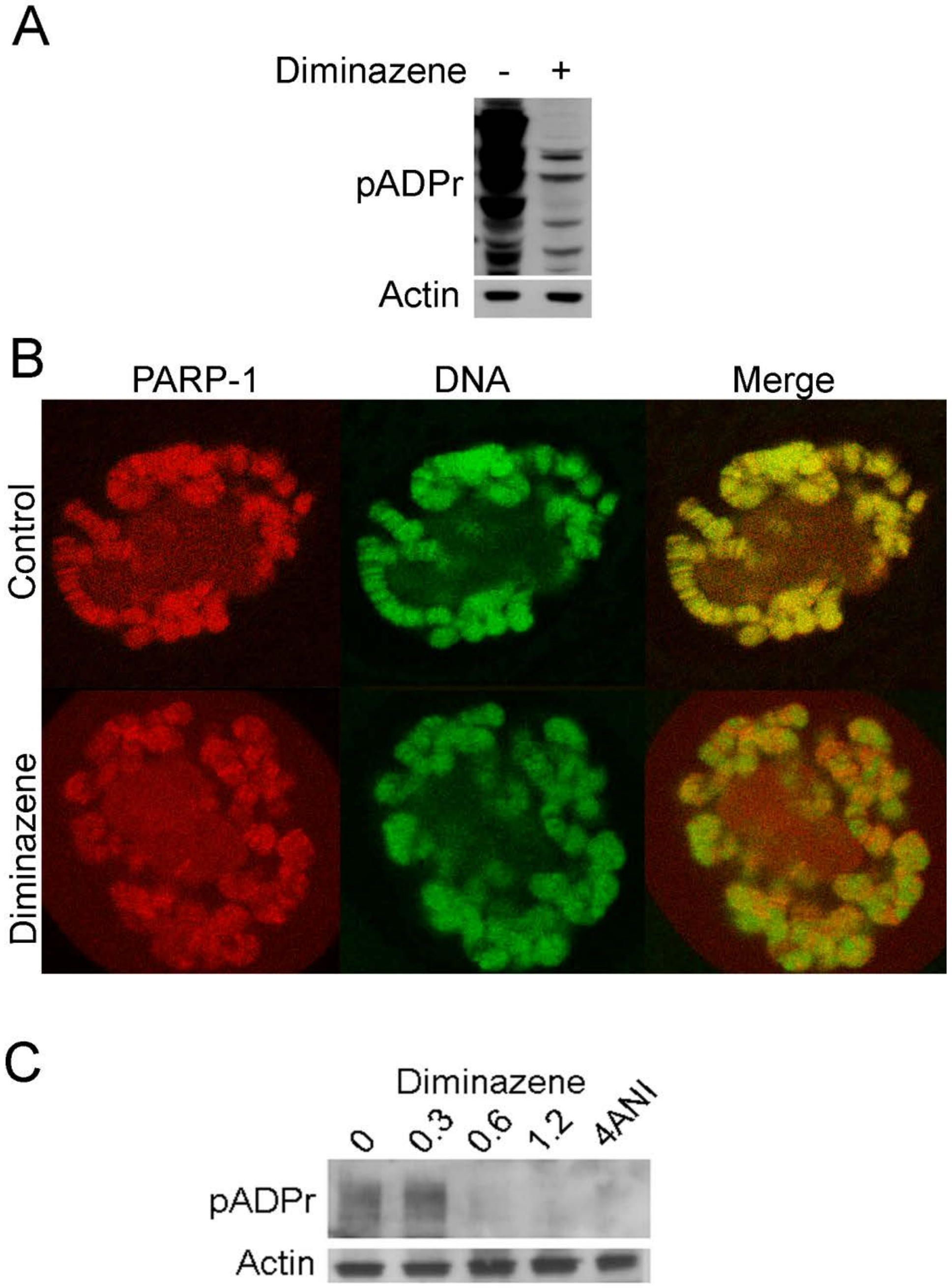

Figure S2. Minor groove binding molecule diminazene disrupts DNA-dependent PARP-1 localization and functions *in vivo* in *Drosophila*. **A.** A comparative analysis of PAPR-1 protein activity in the $parg^{27.1}$ mutant third-instar larvae cultured with or without diminazene in the media. To detect pADPr on Western blot, mAb 10H antibody against pADPr was used. pAb antibody against Actin was used as a loading control. **B.** The treatment with diminazene disrupts DNA-dependent PARP-1 localization in heterochromatin of *Drosophila*. Comparison of PARP-1 protein (red) localization in salivary gland polytene nuclei in untreated control animals and after 39 hrs of culturing with diminazene. **C**. A comparative analysis of PARP protein activity in cancer cells cultured with or without 4ANI or diminazene (0-1.2μM). To detect pADPr on Western blot, mAb 10H antibody against pADPr was used. pAb antibody against Actin was used as a loading control.

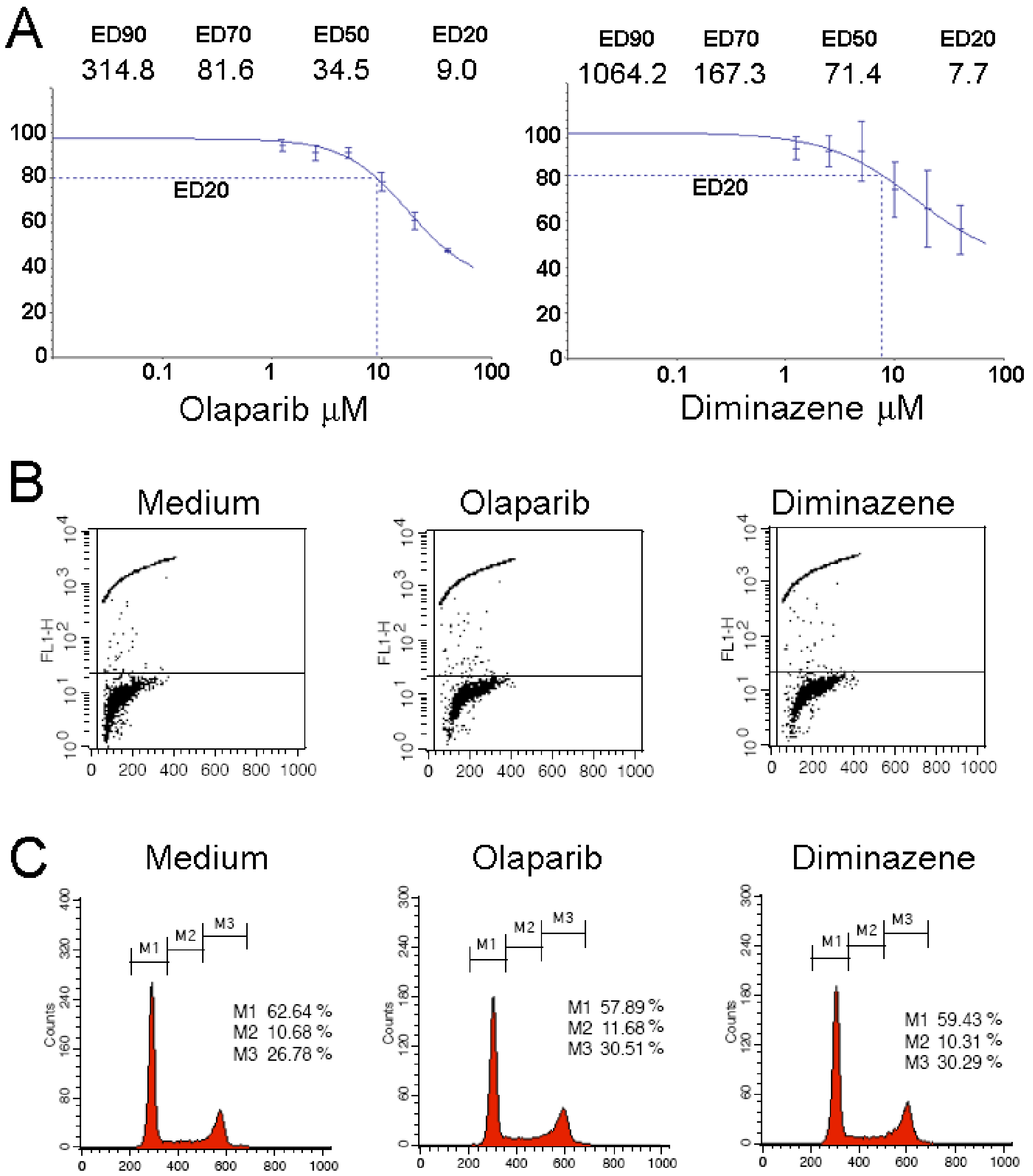

Figure S3. PARP-1 inhibitors do not suppress proliferation of cancer cells. A. Estimated concentration of effective doses (ED) for PARP-1 inhibitors Olaparib and Diminazene. PNX cells were cultured with various concentrations of inhibitors for 96 hrs. Cellular proliferation was assessed using the CellTiter-Blue assay. The effective doses (ED) were calculated using XLfit software. **B.** DNA fragmentation assay. PNX cells were treated with Olaparib or diminazene (10uM) for 48 hrs. The percentage of apoptotic cells was determined by TUNEL assay followed by flow cytometry analysis. **C.** Cell cycle analysis. Aliquots of samples were treated as described in panel **B**, stained with PI and analyzed by flow cytometry. Numbers represent percentage of cells in G1, S and G2/M phases of cell cycle, respectively.

SUPPLEMENTAL MATERIALS AND METHODS

In vitro measurement of cell proliferation.

Cell proliferation was determined by CellTiter-Blue assay (Promega, Madison, WI). Effects of MGBLs and classical PARP-1 inhibitors on cellular proliferation were assessed as published previously using the CellTiter-Blue assay. Both types of small molecules have minor effect on cell proliferation in PNX cells. The CellTiter-Blue assay is based on the ability of living cells to convert a redox dye (resazurin) into a fluorescent end product (resorufin). The divergence in the activity between MGBLs and classical PARP-1 inhibitors is further highlighted by the calculated range of effective doses (ED) for both compounds against the PNX cell line, as displayed in Supplemental Figure S3.

Clonogenic Cell Survival Assay

Cells were plated into 24-well plates at a density of 2000cells/well. Cells were allowed to adhere overnight at 37°C, followed by treatment with increasing concentrations of diminazene and Olaparib for 14 days. Colonies were fixed with 70% ethanol for 10 min and stained with 0.25% methylene blue in 30% ethanol for 10 min. After that, staining solution was removed, and plates were rinsed with water. Colonies consisting of 50 cells or more were counted. Plating efficiencies (PE) were calculated as follows: PE = number of colonies/number of cells seeded. The surviving fraction (SF) was calculated as follows: SF = number of colonies/number of cells seeded × PE.

Antibodies

The following antibodies were used: rabbit polyclonal anti-PARP-1 (C2-10, Trevigen), anti-H2Av (gift from Dr. R. Glaser), anti-B-Actin (Mouse monoclonal, Sigma, #A5441) and anti-pADPr (Mouse monoclonal, Tulip, #1020). Either goat anti-rabbit or anti-mouse secondary antibodies were conjugated to horseradish peroxidase (Sigma).

Quantitative analysis of RNA

Total RNA was purified according to manufacturer's instructions (Qiagen). cDNA was synthesized from 2 μg of purified total RNA (Invitrogen). qPCR was performed on a StepOnePlus™ Applied Biosystems Real-Time PCR System, using 2x SYBR® Green Master PCR Mix according to the manufacturer's instructions (both from Applied Biosystems, Foster City, CA, USA). The primer sequences used in RT-qPCR are available on request. All amplifications were performed in triplicate using 2.0 μL of cDNA per reaction. Triplicate mean values were calculated according to the ΔΔCt quantification method using *Actin* gene transcription as a reference for normalization. Changes in expression were quantitated by the ΔΔ threshold cycle (ΔΔCt) method as described (Livak et al., 2001). For primary transcript analysis, purified total RNA was treated by Deoxyribonuclease I (Qiagen). Product accumulation was monitored by SYBR green fluorescence and normalized *Actin* mRNA. Control reactions lacking reverse transcriptase yielded little to no signal.

Western blotting

For semi-quantitative protein analysis, whole extracts were prepared by boiling cells or homogenized third-instar larvae for 10 min in SDS sample buffer [25 mM Tris (pH 6.8), 2% β-mercaptoethanol, 3% SDS, 0.1% bromophenol blue, 5% glycerol] at 1 x 10^7 cells/ml, and proteins were resolved by SDS-PAGE and transferred to i-Blot (Invitrogen). Detection was performed with ECL-Plus (Amersham) and HyBlot CL Autoradiography Film. Image digitizing and quantitative analysis were performed by Odyssey v1.2 software (LI-COR, Lincoln, NE).

PARP-1 activity assay

1 µl of H4-histone (1γ/µl) or endonuclease-digested plasmid DNA (0.01 γ/ µl) was mixed with 25 µl 200µM NAD and 1 µl of inhibitor/water. This mixture was combined with 10xPARP-1 reaction buffer (500 mM Tris, pH8.0, 250 mM MgCl2, 1%Triton X-100) and 0.7 µl PARP-1 enzyme (0.1γ/ µl, Trevigen). All the reactions were carried out for 30 min at room temperature. Samples were examined with SDS-PAGE and Western Blot using anti-pADPr antibody.

***In vitro* interaction assay**

H4-histones were isolated according to the protocol of Luger and co-authors (Luger et al., 1999). Protein coupling to CnBr-activated Sepharose beads (GE Healthcare) and *in vitro* binding assay were performed as described previously (Cirillo et al., 2002). Beads coupled to H4 histone or DNA were washed once for ten minutes in washing/binding buffer (10mM Tris-HCl, pH8.0, 140mM NaCl, 3mM DTT, and 0.1%Triton X-100) at room temperature with gentle rotation. Washed beads were divided into 20 µl samples and incubated with Hoechst^{33342} at different concentrations for 1min at room temperature. Then 3 µl of PARP-1 enzyme (0.1γ/ µl, Trevigen) were added, and the mixture was incubated for 20 min at room temperature with gentle rotation. All the samples were centrifuged for 1 min at 1500 rpm, RT. The supernatant was removed and put onto ice, while the pellet was washed 3 times in washing/binding buffer (10 min, RT, gentle rotation). Samples were examined with SDS-PAGE and Western Blot using anti-PARP-1 antibody.

***In vitro* binding – activation assay**

Washed protein/DNA coupled beads (see *in vitro* interaction assay) were divided into 20 µl samples and incubated with Hoechst^{33342} at different concentrations for 1min at room temperature. Then 3 µl of PARP-1 enzyme (0.1γ/ µl, Trevigen) were added, and the mixture was incubated for 20 min at room temperature with gentle rotation. All the samples were centrifuged for 1 min at 1500 rpm, RT. The supernatant (S) was removed and put onto ice, while the pellet was washed 3 times in 1xPARP-1 reaction buffer (10 min, RT, gentle rotation). This sample was divided into two aliquots: P1 and P2. Sample P1 was put onto ice. P2 was mixed with 25 µl 200µM NAD and 1 µl of Hoechst^{33342}/water. This mixture was combined with 10x PARP-1 reaction buffer (PARP-1 activity assay) and 0.3γ PARP-1 enzyme. The reaction was carried out for 30 min at room temperature. The samples were examined with SDS-PAGE and Western Blot using anti-PARP antibody (for S and P1) and anti-pADPr antibody (for P2).

PARP-1 inhibition *in vivo* in *Drosophila*

The fly stocks were generated by the standard genetic methods or obtained from the Bloomington *Drosophila* Stock Center and the Exelixis Collection at the Harvard Medical School, except as indicated. Genetic markers are described in Flybase (www.flybase.org). pP{w1, UAS::PARP-1-DsRed} was described by Tulin et al. (2002). *parg*$^{27.1}$ flies (Hanai et al., 2004; Kotova et al., 2010) were mass mated in the vials with standard corn medium. After 4 days, parents were removed from the vials, and different doses of Hoechst were mixed into the fly food, containing asynchronous *parg*$^{27.1}$ embryos (dose - 330 µl of 10mM Hoechst^{33342} per each 8.25 g of medium). After 16 or 39 hrs, homozygous *parg*$^{27.1}$ mature third-instar larvae were collected and homogenized. Protein samples were examined with SDS-PAGE and Western Blot using anti-pADPr antibody.

Confocal microscopy was performed as described (Kotova et al., 2010).

ChIP Assay

WT flies were mass mated in vials with standard corn medium. In 4 days, 330 µl of 20mM Hoechst^{33342} per each 8.25 g of medium were mixed into the food, containing asynchronous WT embryos. After 48 hrs, mature third-instar larvae were collected, crosslinked in 4% formaldehyde (30 min) and rinsed three times in physiological brine. Then larvae were ground

in lysis buffer [50mM Tris-HCl (pH8.0), 300mM NaCl, 1% Nonidet P-40, 1mM EDTA, 1mM DTT and 1x protease inhibitor mixture (Roche)], and sonication was performed using a BioRuptor sonicator (Diagenode) to produce fragments of approximately 500 bp. The samples were processed for ChIP assay kit (Millipore), following the manufacturer's instructions. All protocols were carried out as described previously (Kotova et al., 2010).

Molecular modeling

Using Chimera software (Pettersen et al., 2004), we superimposed two previously reported crystal structures of Hoechst^{33342}-DNA (Sriram et al., 1992) (PDB Code 129D) and PARP-1 Zn-finger-DNA complex (Ali et al., 2012) (PDB Code 4AV1). Since Hoechst^{33342} was shown to interact with the central A:T base pairs in this particular duplex DNA, these base pairs were aligned and superimposed on the base pairs shown to interact with PARP Znf2. The DNA surface representation was done in Chimera using the MSMS package (Sanner et al., 1996), and the ray-traced image was prepared with POV-Ray (Persistence of Vision Pty. Ltd. 2004).

Mouse xenograph model

For in vivo studies, 1 x 106 PC-3 cells were inoculated s.c. in the flank region of 6 week-old male C.B17/Icr-scid mice using a 27-gauge needle. Six- to eight-week-old C.B17/Icr-scid mice were used. Mice were selected on the basis of adequate body weight gain and freedom from clinical signs of disease or injury. Animals were maintained in microisolator cages with controlled environment (21-23°C) in a designated pathogen-free colony and provided food pellets (AIN-93M diet) and water ad libitum. Ten days after the injection of tumor cells, animals were randomly assigned to the control or experimental groups (n=5 mice/group). The mice were treated with the MGBL diminazene (23 mg/kg) or vehicle (0.9% NaCl) and classical PARP1 inhibitor Olaparib (50mg/kg) or vehicle (PBS + 10% (2-Hydroxypropil)-b-cyclodextrin). Mice were sacrificed by CO2 asphyxiation 3-4 weeks after tumor transplantation or before discomfort occurs (i.e., animals whose tumor load reaches l0% of body weight, animals with tumors which have ulcerated through the skin, or animals judged by appearance, behavior, or clinical signs to be in severe pain, distress or near death).

SUPPLEMENTAL REFERENCES

Ali AA, Timinszky G, Arribas-Bosacoma R, Kozlowski M, Hassa PO, Hassler M, Ladurner AG, Pearl LH, Oliver AW. The zinc-finger domains of PARP1 cooperate to recognize DNA strand breaks. Nat Struct Mol Biol. 2012; 19: 685.

Cirillo LA, et al. Opening of compacted chromatin by early developmental transcription factors HNF3 (FoxA) and GATA-4. Mol Cell. 2002; 9: 279-289.

Hanai S, et al. Loss of poly(ADP-ribose) glycohydrolase causes progressive neurodegeneration in Drosophila melanogaster. Proc Natl Acad Sci USA. 2004; 101: 82–86.

Livak KJ, Schmittgen TD. Analysis of relative gene expression data using real-time quantitative PCR and the 2(-Delta Delta C(T)). Method Methods. 2001; 25: 402-408.

Luger K, Rechsteiner TJ, Richmond TJ. Expression and purification of recombinant histones and nucleosome reconstitution. Methods Mol Biol. 1999; 119: 1-16.

Persistence of Vision Pty Ltd. Persistence of Vision Raytracer (Version 3.6) [Computer software]. 2004. Retrieved from http://www.povray.org/download/

Pettersen EF, Goddard TD, Huang CC, Couch GS, Greenblatt DM, Meng EC, Ferrin TE. UCSF Chimera--a visualization system for exploratory research and analysis. J Comput Chem. 2004; 25(13): 1605-1612.

Sanner MF, Olson AJ, Spehner JC. Reduced surface: an efficient way to compute molecular surfaces. Biopolymers. 1996; 38(3): 305-320.
